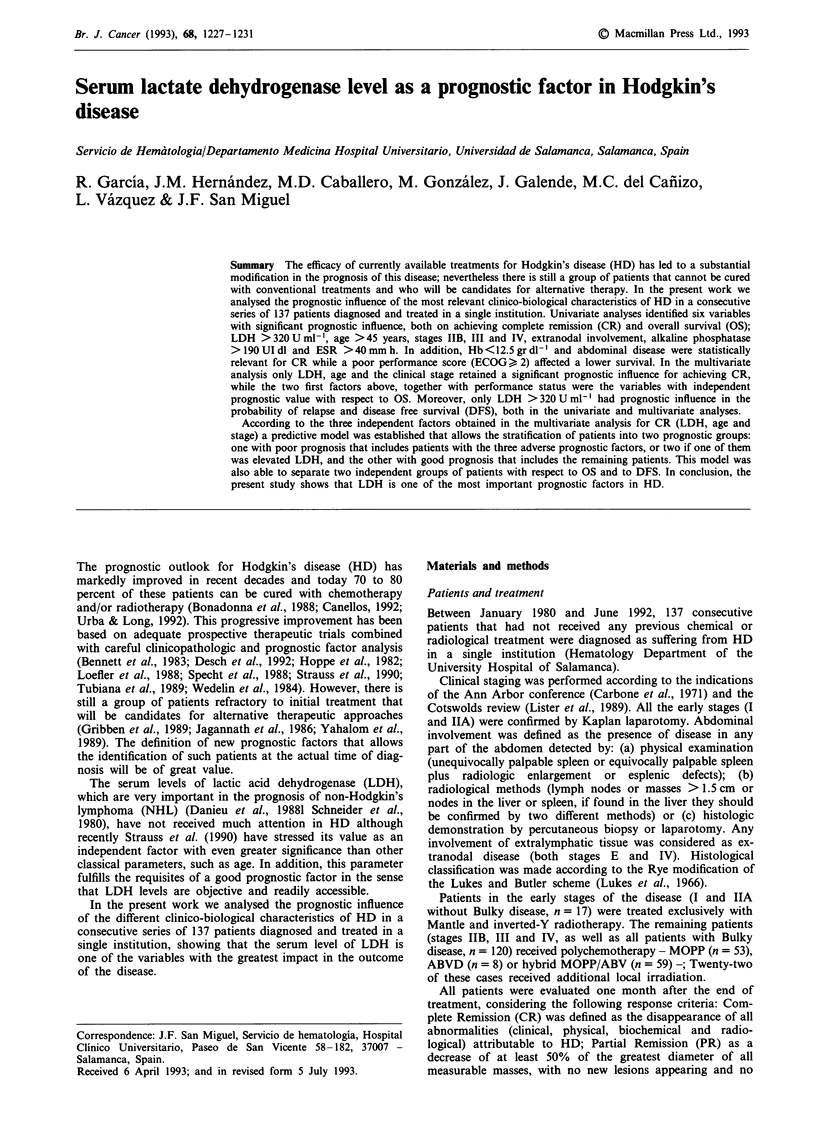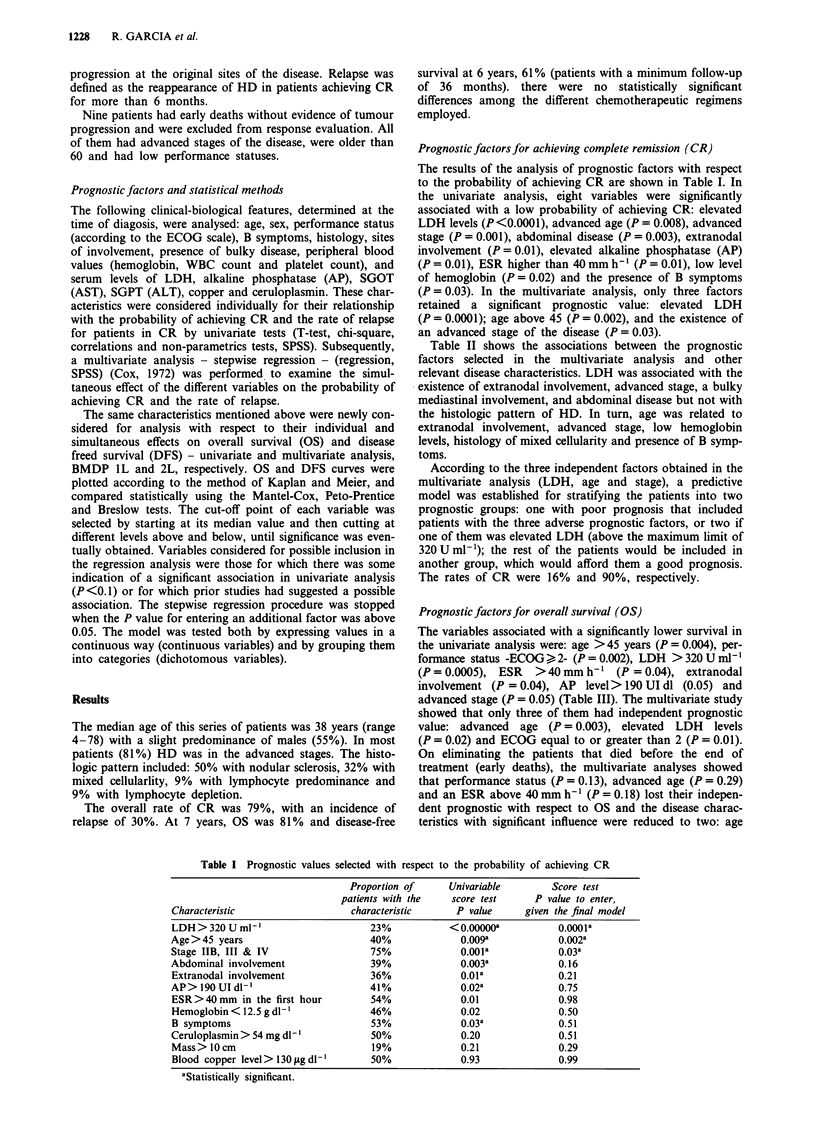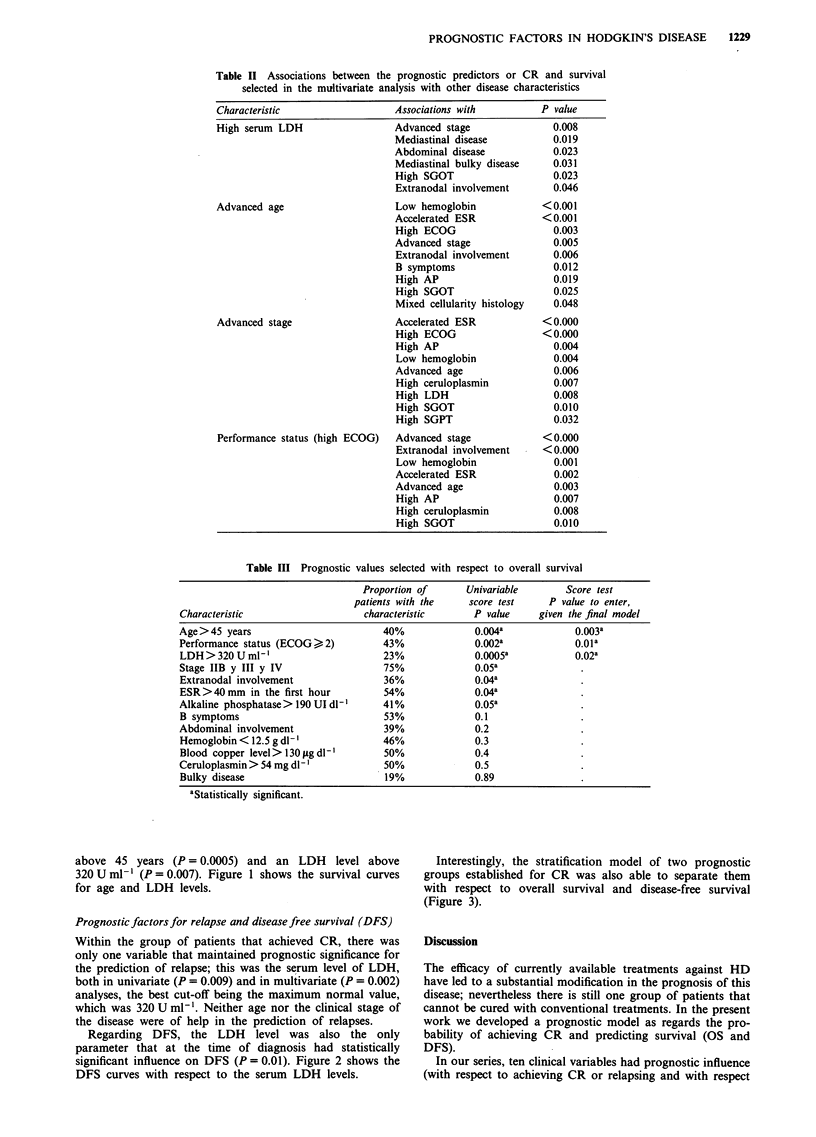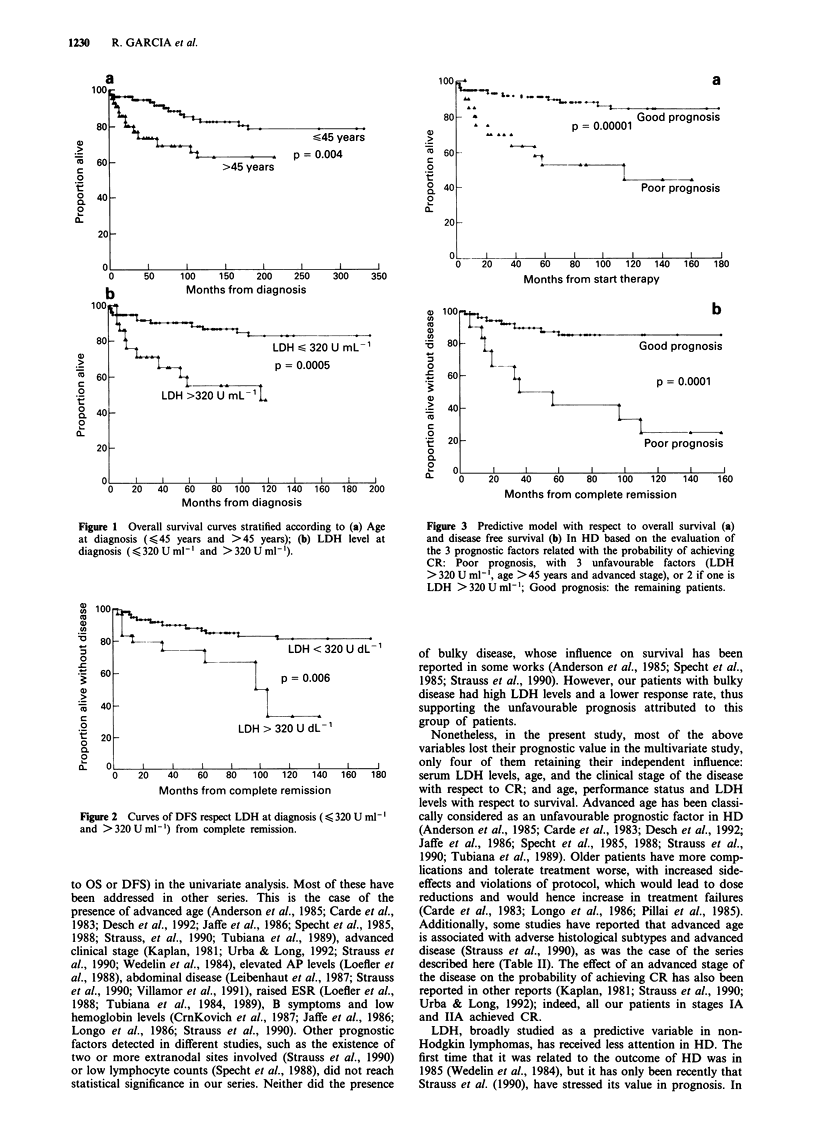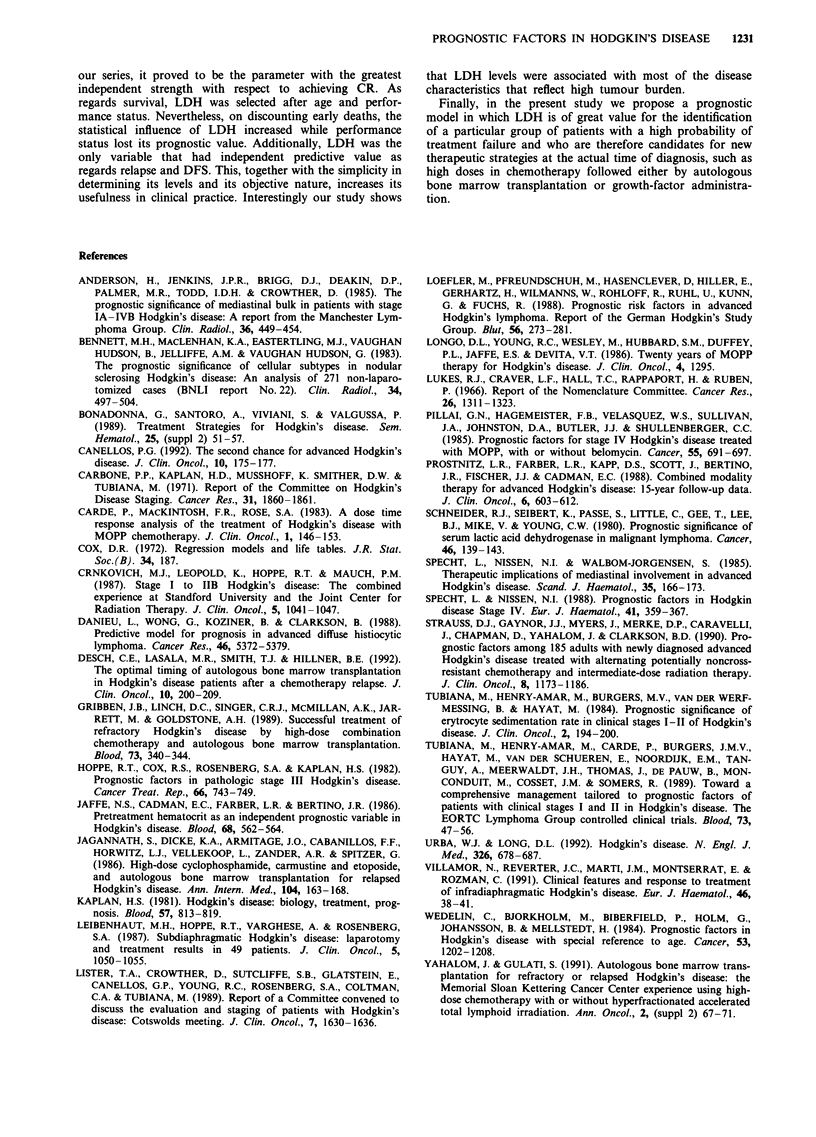# Serum lactate dehydrogenase level as a prognostic factor in Hodgkin's disease.

**DOI:** 10.1038/bjc.1993.509

**Published:** 1993-12

**Authors:** R. García, J. M. Hernández, M. D. Caballero, M. González, J. Galende, M. C. del Cañizo, L. Vázquez, J. F. San Miguel

**Affiliations:** Servicio de Hemàtologia/Departamento Medicina Hospital Universitario, Universidad de Salamanca, Spain.

## Abstract

The efficacy of currently available treatments for Hodgkin's disease (HD) has led to a substantial modification in the prognosis of this disease; nevertheless there is still a group of patients that cannot be cured with conventional treatments and who will be candidates for alternative therapy. In the present work we analysed the prognostic influence of the most relevant clinico-biological characteristics of HD in a consecutive series of 137 patients diagnosed and treated in a single institution. Univariate analyses identified six variables with significant prognostic influence, both on achieving complete remission (CR) and overall survival (OS); LDH > 320 U ml-1, age > 45 years, stages IIB, III and IV, extranodal involvement, alkaline phosphatase > 190 UI dl and ESR > 40 mm h. In addition, Hb < 12.5 gr dl-1 and abdominal disease were statistically relevant for CR while a poor performance score (ECOG > or = 2) affected a lower survival. In the multivariate analysis only LDH, age and the clinical stage retained a significant prognostic influence for achieving CR, while the two first factors above, together with performance status were the variables with independent prognostic value with respect to OS. Moreover, only LDH > 320 U ml-1 had prognostic influence in the probability of relapse and disease free survival (DFS), both in the univariate and multivariate analyses. According to the three independent factors obtained in the multivariate analysis for CR (LDH, age and stage) a predictive model was established that allows the stratification of patients into two prognostic groups: one with poor prognosis that includes patients with the three adverse prognostic factors, or two if one of them was elevated LDH, and the other with good prognosis that includes the remaining patients. This model was also able to separate two independent groups of patients with respect to OS and to DFS. In conclusion, the present study shows that LDH is one of the most important prognostic factors in HD.


Br. J. Cancer (1993), 68, 1227-1231 © Macmillan Press Ltd., 1993

### Serum lactate dehydrogenase level as a prognostic factor in Hodgkin's disease

*Servicio de Hemàtologia/Departamento Medicina Hospital Universitario, Universidad de Salamanca, Salamanca, Spain*

R. García, J.M. Hernández, M.D. Caballero, M. González, J. Galende, M.C. del Cañizo, L. Vázquez & J.F. San Miguel

**Summary** The efficacy of currently available treatments for Hodgkin's disease (HD) has led to a substantial modification in the prognosis of this disease; nevertheless there is still a group of patients that cannot be cured with conventional treatments and who will be candidates for alternative therapy. In the present work we analysed the prognostic influence of the most relevant clinico-biological characteristics of HD in a consecutive series of 137 patients diagnosed and treated in a single institution. Univariate analyses identified six variables with significant prognostic influence, both on achieving complete remission (CR) and overall survival (OS); LDH $>320\,U\,ml^{-1}$, age $>45$ years, stages IIB, III and IV, extranodal involvement, alkaline phosphatase $>190$ UI dl and ESR $>40$ mm h. In addition, Hb$<12.5\,gr\,dl^{-1}$ and abdominal disease were statistically relevant for CR while a poor performance score (ECOG$\geqslant 2$) affected a lower survival. In the multivariate analysis only LDH, age and the clinical stage retained a significant prognostic influence for achieving CR, while the two first factors above, together with performance status were the variables with independent prognostic value with respect to OS. Moreover, only LDH $>320\,U\,ml^{-1}$ had prognostic influence in the probability of relapse and disease free survival (DFS), both in the univariate and multivariate analyses.

According to the three independent factors obtained in the multivariate analysis for CR (LDH, age and stage) a predictive model was established that allows the stratification of patients into two prognostic groups: one with poor prognosis that includes patients with the three adverse prognostic factors, or two if one of them was elevated LDH, and the other with good prognosis that includes the remaining patients. This model was also able to separate two independent groups of patients with respect to OS and to DFS. In conclusion, the present study shows that LDH is one of the most important prognostic factors in HD.

The prognostic outlook for Hodgkin's disease (HD) has markedly improved in recent decades and today 70 to 80 percent of these patients can be cured with chemotherapy and/or radiotherapy (Bonadonna *et al.*, 1988; Canellos, 1992; Urba & Long, 1992). This progressive improvement has been based on adequate prospective therapeutic trials combined with careful clinicopathologic and prognostic factor analysis (Bennett *et al.*, 1983; Desch *et al.*, 1992; Hoppe *et al.*, 1982; Loefler *et al.*, 1988; Specht *et al.*, 1988; Strauss *et al.*, 1990; Tubiana *et al.*, 1989; Wedelin *et al.*, 1984). However, there is still a group of patients refractory to initial treatment that will be candidates for alternative therapeutic approaches (Gribben *et al.*, 1989; Jagannath *et al.*, 1986; Yahalom *et al.*, 1989). The definition of new prognostic factors that allows the identification of such patients at the actual time of diagnosis will be of great value.

The serum levels of lactic acid dehydrogenase (LDH), which are very important in the prognosis of non-Hodgkin's lymphoma (NHL) (Danieu *et al.*, 19881 Schneider *et al.*, 1980), have not received much attention in HD although recently Strauss *et al.* (1990) have stressed its value as an independent factor with even greater significance than other classical parameters, such as age. In addition, this parameter fulfills the requisites of a good prognostic factor in the sense that LDH levels are objective and readily accessible.

In the present work we analysed the prognostic influence of the different clinico-biological characteristics of HD in a consecutive series of 137 patients diagnosed and treated in a single institution, showing that the serum level of LDH is one of the variables with the greatest impact in the outcome of the disease.

Correspondence: J.F. San Miguel, Servicio de hematología, Hospital Clínico Universitario, Paseo de San Vicente 58-182, 37007 - Salamanca, Spain.
Received 6 April 1993; and in revised form 5 July 1993.

### Materials and methods

### *Patients and treatment*

Between January 1980 and June 1992, 137 consecutive patients that had not received any previous chemical or radiological treatment were diagnosed as suffering from HD in a single institution (Hematology Department of the University Hospital of Salamanca).

Clinical staging was performed according to the indications of the Ann Arbor conference (Carbone *et al.*, 1971) and the Cotswolds review (Lister *et al.*, 1989). All the early stages (I and IIA) were confirmed by Kaplan laparotomy. Abdominal involvement was defined as the presence of disease in any part of the abdomen detected by: (a) physical examination (unequivocally palpable spleen or equivocally palpable spleen plus radiologic enlargement or esplenic defects); (b) radiological methods (lymph nodes or masses $>1.5$ cm or nodes in the liver or spleen, if found in the liver they should be confirmed by two different methods) or (c) histologic demonstration by percutaneous biopsy or laparotomy. Any involvement of extralymphatic tissue was considered as extranodal disease (both stages E and IV). Histological classification was made according to the Rye modification of the Lukes and Butler scheme (Lukes *et al.*, 1966).

Patients in the early stages of the disease (I and IIA without Bulky disease, $n = 17$) were treated exclusively with Mantle and inverted-Y radiotherapy. The remaining patients (stages IIB, III and IV, as well as all patients with Bulky disease, $n = 120$) received polychemotherapy - MOPP ($n = 53$), ABVD ($n = 8$) or hybrid MOPP/ABV ($n = 59$) -; Twenty-two of these cases received additional local irradiation.

All patients were evaluated one month after the end of treatment, considering the following response criteria: Complete Remission (CR) was defined as the disappearance of all abnormalities (clinical, physical, biochemical and radiological) attributable to HD; Partial Remission (PR) as a decrease of at least 50% of the greatest diameter of all measurable masses, with no new lesions appearing and no progression at the original sites of the disease. Relapse was defined as the reappearance of HD in patients achieving CR for more than 6 months.

Nine patients had early deaths without evidence of tumour progression and were excluded from response evaluation. All of them had advanced stages of the disease, were older than 60 and had low performance statuses.

*Prognostic factors and statistical methods*

The following clinical-biological features, determined at the time of diagosis, were analysed: age, sex, performance status (according to the ECOG scale), B symptoms, histology, sites of involvement, presence of bulky disease, peripheral blood values (hemoglobin, WBC count and platelet count), and serum levels of LDH, alkaline phosphatase (AP), SGOT (AST), SGPT (ALT), copper and ceruloplasmin. These characteristics were considered individually for their relationship with the probability of achieving CR and the rate of relapse for patients in CR by univariate tests (T-test, chi-square, correlations and non-parametrics tests, SPSS). Subsequently, a multivariate analysis – stepwise regression – (regression, SPSS) (Cox, 1972) was performed to examine the simultaneous effect of the different variables on the probability of achieving CR and the rate of relapse.

The same characteristics mentioned above were newly considered for analysis with respect to their individual and simultaneous effects on overall survival (OS) and disease freed survival (DFS) – univariate and multivariate analysis, BMDP 1L and 2L, respectively. OS and DFS curves were plotted according to the method of Kaplan and Meier, and compared statistically using the Mantel-Cox, Peto-Prentice and Breslow tests. The cut-off point of each variable was selected by starting at its median value and then cutting at different levels above and below, until significance was eventually obtained. Variables considered for possible inclusion in the regression analysis were those for which there was some indication of a significant association in univariate analysis ($P<0.1$) or for which prior studies had suggested a possible association. The stepwise regression procedure was stopped when the $P$ value for entering an additional factor was above 0.05. The model was tested both by expressing values in a continuous way (continuous variables) and by grouping them into categories (dichotomous variables).

### Results

The median age of this series of patients was 38 years (range 4–78) with a slight predominance of males (55%). In most patients (81%) HD was in the advanced stages. The histologic pattern included: 50% with nodular sclerosis, 32% with mixed cellularlity, 9% with lymphocyte predominance and 9% with lymphocyte depletion.

The overall rate of CR was 79%, with an incidence of relapse of 30%. At 7 years, OS was 81% and disease-free survival at 6 years, 61% (patients with a minimum follow-up of 36 months). there were no statistically significant differences among the different chemotherapeutic regimens employed.

*Prognostic factors for achieving complete remission (CR)*

The results of the analysis of prognostic factors with respect to the probability of achieving CR are shown in Table I. In the univariate analysis, eight variables were significantly associated with a low probability of achieving CR: elevated LDH levels ($P<0.0001$), advanced age ($P=0.008$), advanced stage ($P=0.001$), abdominal disease ($P=0.003$), extranodal involvement ($P=0.01$), elevated alkaline phosphatase (AP) ($P=0.01$), ESR higher than 40 mm h$^{-1}$ ($P=0.01$), low level of hemoglobin ($P=0.02$) and the presence of B symptoms ($P=0.03$). In the multivariate analysis, only three factors retained a significant prognostic value: elevated LDH ($P=0.0001$); age above 45 ($P=0.002$), and the existence of an advanced stage of the disease ($P=0.03$).

Table II shows the associations between the prognostic factors selected in the multivariate analysis and other relevant disease characteristics. LDH was associated with the existence of extranodal involvement, advanced stage, a bulky mediastinal involvement, and abdominal disease but not with the histologic pattern of HD. In turn, age was related to extranodal involvement, advanced stage, low hemoglobin levels, histology of mixed cellularity and presence of B symptoms.

According to the three independent factors obtained in the multivariate analysis (LDH, age and stage), a predictive model was established for stratifying the patients into two prognostic groups: one with poor prognosis that included patients with the three adverse prognostic factors, or two if one of them was elevated LDH (above the maximum limit of 320 U ml$^{-1}$); the rest of the patients would be included in another group, which would afford them a good prognosis. The rates of CR were 16% and 90%, respectively.

*Prognostic factors for overall survival (OS)*

The variables associated with a significantly lower survival in the univariate analysis were: age $>45$ years ($P=0.004$), performance status -ECOG$\geqslant$2- ($P=0.002$), LDH $>320$ U ml$^{-1}$ ($P=0.0005$), ESR $>40$ mm h$^{-1}$ ($P=0.04$), extranodal involvement ($P=0.04$), AP level$>$190 UI dl (0.05) and advanced stage ($P=0.05$) (Table III). The multivariate study showed that only three of them had independent prognostic value: advanced age ($P=0.003$), elevated LDH levels ($P=0.02$) and ECOG equal to or greater than 2 ($P=0.01$). On eliminating the patients that died before the end of treatment (early deaths), the multivariate analyses showed that performance status ($P=0.13$), advanced age ($P=0.29$) and an ESR above 40 mm h$^{-1}$ ($P=0.18$) lost their independent prognostic with respect to OS and the disease characteristics with significant influence were reduced to two: age

**Table I** Prognostic values selected with respect to the probability of achieving CR

| *Characteristic* | *Proportion of patients with the characteristic* | *Univariable score test P value* | *Score test P value to enter, given the final model* |
|---|---|---|---|
| LDH > 320 U ml$^{-1}$ | 23% | < 0.00000[a] | 0.0001[a] |
| Age > 45 years | 40% | 0.009[a] | 0.002[a] |
| Stage IIB, III & IV | 75% | 0.001[a] | 0.03[a] |
| Abdominal involvement | 39% | 0.003[a] | 0.16 |
| Extranodal involvement | 36% | 0.01[a] | 0.21 |
| AP > 190 UI dl$^{-1}$ | 41% | 0.02[a] | 0.75 |
| ESR > 40 mm in the first hour | 54% | 0.01 | 0.98 |
| Hemoglobin < 12.5 g dl$^{-1}$ | 46% | 0.02 | 0.50 |
| B symptoms | 53% | 0.03[a] | 0.51 |
| Ceruloplasmin > 54 mg dl$^{-1}$ | 50% | 0.20 | 0.51 |
| Mass > 10 cm | 19% | 0.21 | 0.29 |
| Blood copper level > 130 μg dl$^{-1}$ | 50% | 0.93 | 0.99 |

[a]Statistically significant.

**Table II** Associations between the prognostic predictors or CR and survival selected in the multivariate analysis with other disease characteristics

| *Characteristic* | *Associations with* | *P value* |
|---|---|---|
| High serum LDH | Advanced stage | 0.008 |
| | Mediastinal disease | 0.019 |
| | Abdominal disease | 0.023 |
| | Mediastinal bulky disease | 0.031 |
| | High SGOT | 0.023 |
| | Extranodal involvement | 0.046 |
| Advanced age | Low hemoglobin | <0.001 |
| | Accelerated ESR | <0.001 |
| | High ECOG | 0.003 |
| | Advanced stage | 0.005 |
| | Extranodal involvement | 0.006 |
| | B symptoms | 0.012 |
| | High AP | 0.019 |
| | High SGOT | 0.025 |
| | Mixed cellularity histology | 0.048 |
| Advanced stage | Accelerated ESR | <0.000 |
| | High ECOG | <0.000 |
| | High AP | 0.004 |
| | Low hemoglobin | 0.004 |
| | Advanced age | 0.006 |
| | High ceruloplasmin | 0.007 |
| | High LDH | 0.008 |
| | High SGOT | 0.010 |
| | High SGPT | 0.032 |
| Performance status (high ECOG) | Advanced stage | <0.000 |
| | Extranodal involvement | <0.000 |
| | Low hemoglobin | 0.001 |
| | Accelerated ESR | 0.002 |
| | Advanced age | 0.003 |
| | High AP | 0.007 |
| | High ceruloplasmin | 0.008 |
| | High SGOT | 0.010 |

**Table III** Prognostic values selected with respect to overall survival

| *Characteristic* | *Proportion of patients with the characteristic* | *Univariable score test P value* | *Score test P value to enter, given the final model* |
|---|---|---|---|
| Age>45 years | 40% | 0.004[a] | 0.003[a] |
| Performance status (ECOG≥2) | 43% | 0.002[a] | 0.01[a] |
| LDH>320 U ml$^{-1}$ | 23% | 0.0005[a] | 0.02[a] |
| Stage IIB y III y IV | 75% | 0.05[a] | . |
| Extranodal involvement | 36% | 0.04[a] | . |
| ESR>40 mm in the first hour | 54% | 0.04[a] | . |
| Alkaline phosphatase>190 UI dl$^{-1}$ | 41% | 0.05[a] | . |
| B symptoms | 53% | 0.1 | . |
| Abdominal involvement | 39% | 0.2 | . |
| Hemoglobin<12.5 g dl$^{-1}$ | 46% | 0.3 | . |
| Blood copper level>130 μg dl$^{-1}$ | 50% | 0.4 | . |
| Ceruloplasmin>54 mg dl$^{-1}$ | 50% | 0.5 | . |
| Bulky disease | 19% | 0.89 | . |

[a]Statistically significant.

above 45 years ($P = 0.0005$) and an LDH level above 320 U ml$^{-1}$ ($P = 0.007$). Figure 1 shows the survival curves for age and LDH levels.

*Prognostic factors for relapse and disease free survival (DFS)*

Within the group of patients that achieved CR, there was only one variable that maintained prognostic significance for the prediction of relapse; this was the serum level of LDH, both in univariate ($P = 0.009$) and in multivariate ($P = 0.002$) analyses, the best cut-off being the maximum normal value, which was 320 U ml$^{-1}$. Neither age nor the clinical stage of the disease were of help in the prediction of relapses.

Regarding DFS, the LDH level was also the only parameter that at the time of diagnosis had statistically significant influence on DFS ($P = 0.01$). Figure 2 shows the DFS curves with respect to the serum LDH levels.

Interestingly, the stratification model of two prognostic groups established for CR was also able to separate them with respect to overall survival and disease-free survival (Figure 3).

### Discussion

The efficacy of currently available treatments against HD have led to a substantial modification in the prognosis of this disease; nevertheless there is still one group of patients that cannot be cured with conventional treatments. In the present work we developed a prognostic model as regards the probability of achieving CR and predicting survival (OS and DFS).

In our series, ten clinical variables had prognostic influence (with respect to achieving CR or relapsing and with respect

**Figure 1** Overall survival curves stratified according to (**a**) Age at diagnosis (≤45 years and >45 years); (**b**) LDH level at diagnosis (≤320 U $ml^{-1}$ and >320 U $ml^{-1}$).

**Figure 2** Curves of DFS respect LDH at diagnosis (≤320 U $ml^{-1}$ and >320 U $ml^{-1}$) from complete remission.

**Figure 3** Predictive model with respect to overall survival (**a**) and disease free survival (**b**) In HD based on the evaluation of the 3 prognostic factors related with the probability of achieving CR: Poor prognosis, with 3 unfavourable factors (LDH >320 U $ml^{-1}$, age >45 years and advanced stage), or 2 if one is LDH >320 U $ml^{-1}$; Good prognosis: the remaining patients.

to OS or DFS) in the univariate analysis. Most of these have been addressed in other series. This is the case of the presence of advanced age (Anderson *et al.*, 1985; Carde *et al.*, 1983; Desch *et al.*, 1992; Jaffe *et al.*, 1986; Specht *et al.*, 1985, 1988; Strauss, *et al.*, 1990; Tubiana *et al.*, 1989), advanced clinical stage (Kaplan, 1981; Urba & Long, 1992; Strauss *et al.*, 1990; Wedelin *et al.*, 1984), elevated AP levels (Loefler *et al.*, 1988), abdominal disease (Leibenhaut *et al.*, 1987; Strauss *et al.*, 1990; Villamor *et al.*, 1991), raised ESR (Loefler *et al.*, 1988; Tubiana *et al.*, 1984, 1989), B symptoms and low hemoglobin levels (CrnKovich *et al.*, 1987; Jaffe *et al.*, 1986; Longo *et al.*, 1986; Strauss *et al.*, 1990). Other prognostic factors detected in different studies, such as the existence of two or more extranodal sites involved (Strauss *et al.*, 1990) or low lymphocyte counts (Specht *et al.*, 1988), did not reach statistical significance in our series. Neither did the presence of bulky disease, whose influence on survival has been reported in some works (Anderson *et al.*, 1985; Specht *et al.*, 1985; Strauss *et al.*, 1990). However, our patients with bulky disease had high LDH levels and a lower response rate, thus supporting the unfavourable prognosis attributed to this group of patients.

Nonetheless, in the present study, most of the above variables lost their prognostic value in the multivariate study, only four of them retaining their independent influence: serum LDH levels, age, and the clinical stage of the disease with respect to CR; and age, performance status and LDH levels with respect to survival. Advanced age has been classically considered as an unfavourable prognostic factor in HD (Anderson *et al.*, 1985; Carde *et al.*, 1983; Desch *et al.*, 1992; Jaffe *et al.*, 1986; Specht *et al.*, 1985, 1988; Strauss *et al.*, 1990; Tubiana *et al.*, 1989). Older patients have more complications and tolerate treatment worse, with increased side-effects and violations of protocol, which would lead to dose reductions and would hence increase in treatment failures (Carde *et al.*, 1983; Longo *et al.*, 1986; Pillai *et al.*, 1985). Additionally, some studies have reported that advanced age is associated with adverse histological subtypes and advanced disease (Strauss *et al.*, 1990), as was the case of the series described here (Table II). The effect of an advanced stage of the disease on the probability of achieving CR has also been reported in other reports (Kaplan, 1981; Strauss *et al.*, 1990; Urba & Long, 1992); indeed, all our patients in stages IA and IIA achieved CR.

LDH, broadly studied as a predictive variable in non-Hodgkin lymphomas, has received less attention in HD. The first time that it was related to the outcome of HD was in 1985 (Wedelin *et al.*, 1984), but it has only been recently that Strauss *et al.* (1990), have stressed its value in prognosis. In our series, it proved to be the parameter with the greatest independent strength with respect to achieving CR. As regards survival, LDH was selected after age and performance status. Nevertheless, on discounting early deaths, the statistical influence of LDH increased while performance status lost its prognostic value. Additionally, LDH was the only variable that had independent predictive value as regards relapse and DFS. This, together with the simplicity in determining its levels and its objective nature, increases its usefulness in clinical practice. Interestingly our study shows that LDH levels were associated with most of the disease characteristics that reflect high tumour burden.

Finally, in the present study we propose a prognostic model in which LDH is of great value for the identification of a particular group of patients with a high probability of treatment failure and who are therefore candidates for new therapeutic strategies at the actual time of diagnosis, such as high doses in chemotherapy followed either by autologous bone marrow transplantation or growth-factor administration.

### References

ANDERSON, H., JENKINS, J.P.R., BRIGG, D.J., DEAKIN, D.P., PALMER, M.R., TODD, I.D.H. & CROWTHER, D. (1985). The prognostic significance of mediastinal bulk in patients with stage IA–IVB Hodgkin's disease: A report from the Manchester Lymphoma Group. *Clin. Radiol.*, **36**, 449–454.

BENNETT, M.H., MACLENHAN, K.A., EASTERTLING, M.J., VAUGHAN HUDSON, B., JELLIFFE, A.M. & VAUGHAN HUDSON, G. (1983). The prognostic significance of cellular subtypes in nodular sclerosing Hodgkin's disease: An analysis of 271 non-laparotomized cases (BNLI report No. 22). *Clin. Radiol.*, **34**, 497–504.

BONADONNA, G., SANTORO, A., VIVIANI, S. & VALGUSSA, P. (1989). Treatment Strategies for Hodgkin's disease. *Sem. Hematol.*, **25**, (suppl 2) 51–57.

CANELLOS, P.G. (1992). The second chance for advanced Hodgkin's disease. *J. Clin. Oncol.*, **10**, 175–177.

CARBONE, P.P., KAPLAN, H.D., MUSSHOFF, K. SMITHER, D.W. & TUBIANA, M. (1971). Report of the Committee on Hodgkin's Disease Staging. *Cancer Res.*, **31**, 1860–1861.

CARDE, P., MACKINTOSH, F.R., ROSE, S.A. (1983). A dose time response analysis of the treatment of Hodgkin's disease with MOPP chemotherapy. *J. Clin. Oncol.*, **1**, 146–153.

COX, D.R. (1972). Regression models and life tables. *J.R. Stat. Soc.(B)*. **34**, 187.

CRNKOVICH, M.J., LEOPOLD, K., HOPPE, R.T. & MAUCH, P.M. (1987). Stage I to IIB Hodgkin's disease: The combined experience at Standford University and the Joint Center for Radiation Therapy. *J. Clin. Oncol.*, **5**, 1041–1047.

DANIEU, L., WONG, G., KOZINER, B. & CLARKSON, B. (1988). Predictive model for prognosis in advanced diffuse histiocytic lymphoma. *Cancer Res.*, **46**, 5372–5379.

DESCH, C.E., LASALA, M.R., SMITH, T.J. & HILLNER, B.E. (1992). The optimal timing of autologous bone marrow transplantation in Hodgkin's disease patients after a chemotherapy relapse. *J. Clin. Oncol.*, **10**, 200–209.

GRIBBEN, J.B., LINCH, D.C., SINGER, C.R.J., MCMILLAN, A.K., JARRETT, M. & GOLDSTONE, A.H. (1989). Successful treatment of refractory Hodgkin's disease by high-dose combination chemotherapy and autologous bone marrow transplantation. *Blood*, **73**, 340–344.

HOPPE, R.T., COX, R.S., ROSENBERG, S.A. & KAPLAN, H.S. (1982). Prognostic factors in pathologic stage III Hodgkin's disease. *Cancer Treat. Rep.*, **66**, 743–749.

JAFFE, N.S., CADMAN, E.C., FARBER, L.R. & BERTINO, J.R. (1986). Pretreatment hematocrit as an independent prognostic variable in Hodgkin's disease. *Blood*, **68**, 562–564.

JAGANNATH, S., DICKE, K.A., ARMITAGE, J.O., CABANILLOS, F.F., HORWITZ, L.J., VELLEKOOP, L., ZANDER, A.R. & SPITZER, G. (1986). High-dose cyclophosphamide, carmustine and etoposide, and autologous bone marrow transplantation for relapsed Hodgkin's disease. *Ann. Intern. Med.*, **104**, 163–168.

KAPLAN, H.S. (1981). Hodgkin's disease: biology, treatment, prognosis. *Blood*, **57**, 813–819.

LEIBENHAUT, M.H., HOPPE, R.T., VARGHESE, A. & ROSENBERG, S.A. (1987). Subdiaphragmatic Hodgkin's disease: laparotomy and treatment results in 49 patients. *J. Clin. Oncol.*, **5**, 1050–1055.

LISTER, T.A., CROWTHER, D., SUTCLIFFE, S.B., GLATSTEIN, E., CANELLOS, G.P., YOUNG, R.C., ROSENBERG, S.A., COLTMAN, C.A. & TUBIANA, M. (1989). Report of a Committee convened to discuss the evaluation and staging of patients with Hodgkin's disease: Cotswolds meeting. *J. Clin. Oncol.*, **7**, 1630–1636.

LOEFLER, M., PFREUNDSCHUH, M., HASENCLEVER, D, HILLER, E., GERHARTZ, H., WILMANNS, W., ROHLOFF, R., RUHL, U., KUNN, G. & FUCHS, R. (1988). Prognostic risk factors in advanced Hodgkin's lymphoma. Report of the German Hodgkin's Study Group. *Blut*, **56**, 273–281.

LONGO, D.L., YOUNG, R.C., WESLEY, M., HUBBARD, S.M., DUFFEY, P.L., JAFFE, E.S. & DEVITA, V.T. (1986). Twenty years of MOPP therapy for Hodgkin's disease. *J. Clin. Oncol.*, **4**, 1295.

LUKES, R.J., CRAVER, L.F., HALL, T.C., RAPPAPORT, H. & RUBEN, P. (1966). Report of the Nomenclature Committee. *Cancer Res.*, **26**, 1311–1323.

PILLAI, G.N., HAGEMEISTER, F.B., VELASQUEZ, W.S., SULLIVAN, J.A., JOHNSTON, D.A., BUTLER, J.J. & SHULLENBERGER, C.C. (1985). Prognostic factors for stage IV Hodgkin's disease treated with MOPP, with or without belomycin. *Cancer*, **55**, 691–697.

PROSTNITZ, L.R., FARBER, L.R., KAPP, D.S., SCOTT, J., BERTINO, J.R., FISCHER, J.J. & CADMAN, E.C. (1988). Combined modality therapy for advanced Hodgkin's disease: 15-year follow-up data. *J. Clin. Oncol.*, **6**, 603–612.

SCHNEIDER, R.J., SEIBERT, K., PASSE, S., LITTLE, C., GEE, T., LEE, B.J., MIKE, V. & YOUNG, C.W. (1980). Prognostic significance of serum lactic acid dehydrogenase in malignant lymphoma. *Cancer*, **46**, 139–143.

SPECHT, L., NISSEN, N.I. & WALBOM-JORGENSEN, S. (1985). Therapeutic implications of mediastinal involvement in advanced Hodgkin's disease. *Scand. J. Haematol.*, **35**, 166–173.

SPECHT, L. & NISSEN, N.I. (1988). Prognostic factors in Hodgkin disease Stage IV. *Eur. J. Haematol.*, **41**, 359–367.

STRAUSS, D.J., GAYNOR, J.J., MYERS, J., MERKE, D.P., CARAVELLI, J., CHAPMAN, D., YAHALOM, J. & CLARKSON, B.D. (1990). Prognostic factors among 185 adults with newly diagnosed advanced Hodgkin's disease treated with alternating potentially noncross-resistant chemotherapy and intermediate-dose radiation therapy. *J. Clin. Oncol.*, **8**, 1173–1186.

TUBIANA, M., HENRY-AMAR, M., BURGERS, M.V., VAN DER WERF-MESSING, B. & HAYAT, M. (1984). Prognostic significance of erytrocyte sedimentation rate in clinical stages I–II of Hodgkin's disease. *J. Clin. Oncol.*, **2**, 194–200.

TUBIANA, M., HENRY-AMAR, M., CARDE, P., BURGERS, J.M.V., HAYAT, M., VAN DER SCHUEREN, E., NOORDIJK, E.M., TANGUY, A., MEERWALDT, J.H., THOMAS, J., DE PAUW, B., MONCONDUIT, M., COSSET, J.M. & SOMERS, R. (1989). Toward a comprehensive management tailored to prognostic factors of patients with clinical stages I and II in Hodgkin's disease. The EORTC Lymphoma Group controlled clinical trials. *Blood*, **73**, 47–56.

URBA, W.J. & LONG, D.L. (1992). Hodgkin's disease. *N. Engl. J. Med.*, **326**, 678–687.

VILLAMOR, N., REVERTER, J.C., MARTI, J.M., MONTSERRAT, E. & ROZMAN, C. (1991). Clinical features and response to treatment of infradiaphragmatic Hodgkin's disease. *Eur. J. Haematol.*, **46**, 38–41.

WEDELIN, C., BJORKHOLM, M., BIBERFIELD, P., HOLM, G., JOHANSSON, B. & MELLSTEDT, H. (1984). Prognostic factors in Hodgkin's disease with special reference to age. *Cancer*, **53**, 1202–1208.

YAHALOM, J. & GULATI, S. (1991). Autologous bone marrow transplantation for refractory or relapsed Hodgkin's disease: the Memorial Sloan Kettering Cancer Center experience using high-dose chemotherapy with or without hyperfractionated accelerated total lymphoid irradiation. *Ann. Oncol.*, **2**, (suppl 2) 67–71.